# Whole genome sequencing of SARS-CoV-2: evaluating the genetic diversity of variants circulating in Ebonyi State, Southeastern Nigeria

**DOI:** 10.1186/s12879-026-13641-8

**Published:** 2026-05-20

**Authors:** Elizabeth Chibuzo Odeh, Chidinma Stacy Iroha, Ejiro Dowe, Ikechukwu Benjamin Moses, Muhammad Iluoreh Ahmed, Philomena Eromon, Eric Chinonso Nwojiji, Chiedozie Kingsley Ojide, Ezeora Christian Achi, Ifeanyichukwu Romanus Iroha

**Affiliations:** 1 Virology Laboratory, Alex Ekwueme Federal University Teaching Hospital, Abakaliki, Nigeria; 2 Pharmacy Department, Alex Ekwueme Federal University Teaching Hospital, Abakaliki, Nigeria; 3 Department of Pharmaceutical Microbiology and Biotechnology, Faculty of Pharmacy, Delta State University, Abraka, Delta State Nigeria; 4 Department of Applied Microbiology, Faculty of Science, Ebonyi State University, P.M.B. 053, Abakaliki, Nigeria; 5 Department of Molecular Biology and Genomics, African Center of Excellence in Genomics of Infectious Diseases, Ede, Nigeria; 6 Redeemers University, Ede, Nigeria; 7 Ebonyi State Ministry of Health, Abakaliki, Nigeria; 8 College of Medicine, Alex Ekwueme Federal University Ndifu Alike Ikwo, Ikwo, Nigeria

**Keywords:** COVID-19, Whole genome sequencing, Surveillance study, Viral infection, Pandemic

## Abstract

**Background:**

COVID-19 is primarily considered a viral respiratory and vascular illness as its causative agent, SARS-CoV-2, predominantly targets the respiratory and vascular systems. Since being declared a global pandemic by the WHO in 2020, SARS-CoV-2 has spread to 223 countries with more than 281 million cases, and an estimated 5.4 million deaths reported globally. The cardinal aim of this study was to assess the genetic diversity of the circulating SARS-CoV-2 in Ebonyi State, Southeastern Nigeria.

**Methods:**

Over a span of 24 calendar months (April, 2020 to March, 2022), a total of 20 clinical samples (nasopharyngeal, nasal, and oropharyngeal swabs) from individuals who were previously confirmed to be positive for the SAR-COV-2 virus by real-time polymerase chain reaction (qRT-PCR) were randomly selected and submitted for whole genome sequencing using illumina MiSeq sequencer.

**Results:**

Results indicated a very high predominance of SARS-CoV-2 clade 21D (Eta) and pangolin lineage B.1.525, as 19 out of the 20 sequenced strains belonged to this group, while only one belonged to clade 21B and pangolin lineage B.1.1.318.

**Conclusions:**

This study highlighted the dynamics of the circulating SARS-CoV-2 strains in Nigeria. Information from this study will be very crucial in developing and structuring interventions, adaptation of response strategies, and improved preparedness for potential future public health challenges posed by SARS-CoV-2 strains, with a resultant impact of contributing to the broader global effort in combating the pandemic.

## Introduction

A severe respiratory viral infection (COVID-19) of the severe acute respiratory syndrome (SARS-CoV-2) type, caused by the novel coronavirus 2, spread rapidly worldwide, affecting all continents within just four weeks of its emergence in Wuhan, a city in Hubei Province, China, in November 2019 [1].

Since declared a global pandemic, COVID-19 has affected over 13 million people worldwide, resulting in more than 500,000 deaths. Nigeria, one of the severely affected countries, has reported over 33,000 cases and more than 500 fatalities [1, 2]. Nigeria began its COVID-19 vaccination campaign on March 5, 2021, using the Oxford-AstraZeneca vaccine [2]. In Ebonyi State, the introduction of the vaccine helped protect frontline healthcare workers and contributed to a gradual reduction in the spread and mortality rates of COVID-19. However, as noted in various regions in Nigeria, the overall impact of COVID-19 vaccination was initially limited by high vaccine hesitancy and logistical challenges in reaching rural communities. As of July 12, 2020, reports indicated that the coronavirus had spread to over 200 countries across the globe [3]. This global pandemic has prompted many countries to implement lockdown measures that have had a significant impact on the spread of the virus, on social activities and on their respective economies [4].

Studies employing the ideal model which utilized early reported cases from December 2019 to January 2020, such as the work by Majumder and Mandl [5], have estimated the basic reproduction number (R_0_) of SARS-CoV-2 to be within the range of 2.0 to 3.3 [5]. Another study conducted by Wu et al. utilized the susceptible exposed-infected removed (SEIR) model and reported an R_0_ range between 2.47 and 2.86 [6]. For context, previous research on other beta coronaviruses has revealed R_0_ values ranging from 2.2 to 3.6 [7]; thus, suggesting that SARS-CoV-2 exhibits a relatively high level of communicability which has, in turn, contributed to its rapid global dissemination.

COVID-19 typically has an average incubation period of three days [8]. The clinical symptoms of COVID-19 closely resemble those of other viral pneumonias, including fever, fatigue, cough, shortness of breath, and other complications. Severe cases have been associated with organ failure and mortality [1]. These symptoms are more pronounced in adults, often due to underlying chronic conditions such as heart diseases, neurodegenerative disorders, diabetes, or hypertension [9].

Spread of the SARS-CoV-2 virus among humans usually occurs through the inhalation of infected respiratory droplets through the nose or even the mouth. COVID-19 has been noted to have a higher mortality rate, approximately 3.7%, compared to influenza, which typically has a mortality rate of over 1% [1]. Some severe cases of COVID-19 may experience a cytokine storm syndrome and respiratory failure, often due to acute respiratory distress syndrome (ARDS), which is a major cause of death [10]. Similar to other coronaviruses like MERS-CoV and SARS-CoV, there is currently no specific antiviral treatment available for COVID-19. Medical management primarily involves isolation and supportive care, which includes oxygen therapy, fluid management, and antibiotic treatment for secondary bacterial infections.

The lack of clarity regarding the source of the virus, the intricacies of human transmission, and the specific factors influencing its pathogenicity pose significant challenges in developing targeted therapeutic strategies. The absence of a comprehensive assessment of COVID-19 trends in Ebonyi state hinders the formulation of informed policies and impedes the development of strategies to prevent further epidemics and manage infections. While genomic studies have revealed genetic similarities between SARS-CoV-2 and other coronaviruses, critical knowledge gaps persist. This study was designed to assess the genetic diversity of the circulating SARS-CoV-2 in Ebonyi State, Southeastern Nigeria.

## Materials and methods

### Sample collection and processing

This was a cross-sectional study that was carried out for a period of 24 months (April, 2020 to March, 2022) in Ebonyi State, Nigeria. The study subjects included representative samples of individuals living or visiting Ebonyi State. A total of 20 clinical samples (nasopharyngeal, nasal, and oropharyngeal swabs) that were previously confirmed to be positive for the SAR-COV-2 virus by real-time polymerase chain reaction (qRT-PCR) were randomly selected for this study. The kits used to perform assays in the real-time PCR test for the detection of SARS-CoV-2 is the AllPlex TM SARS -COV-2 Plus Variant assay kit (cat No: RV 102932) (Seegene, Inc., South Korea). Collected clinical swab samples were aseptically inserted into viral transport medium (VTM), triple-packaged, and transported (2–4 °C) to a designated NCDC-certified laboratory in Ebonyi State (Virology Lab, AE-FUTHA) and submitted for whole genome sequencing, to determine their epidemiological identities and assess the diversity of SARS-COV-2 variants in circulation in Ebonyi State.

### Viral RNA extraction (spin column protocol)

Total viral RNA was purified from clinical specimens using the QIAamp Viral RNA Mini Kit (QIAGEN) according to the manufacturer’s standardized protocol, optimized for downstream use with the Seegene Allplex™ SARS-CoV-2 assay. Briefly, 140 µL of the homogenized sample in viral transport medium was lysed using 560 µL of buffer containing carrier RNA. Following a 10-minute incubation at room temperature, 560 µL of 99% ethanol was introduced to the lysate to facilitate nucleic acid binding. The mixture was processed through QIAamp mini columns via centrifugation at 8,000 rpm. Systematic washing steps were performed using buffers AW1 and AW2 to remove contaminants. After a final drying centrifugation at 14,000 rpm to eliminate residual ethanol, the purified RNA was eluted in 50 µL of elution buffer and stored for library preparation.

### Library preparation and whole genome sequencing

Sequencing libraries were constructed using the Allplex™ SARS-CoV-2 Panel (Model Genomics, Cat. 102932) for the Illumina platform, utilizing an input range of 10–50 ng of RNA. For quality control, Genomic RNA from SARS-CoV-2 Isolate (VR-1986D) served as a positive control. Library integrity and size distribution were evaluated using the High Sensitivity NGS Fragment Analysis Kit (Advanced Analytical).

A Library Quality Ratio Score (QRS) was calculated by determining the ratio of the target peak (250–350 bp) to the adapter-dimer peak (50–190 bp). Samples achieving a QRS > 1.0 (categorized as “Good” to “Excellent”) were progressed to sequencing. Libraries were denatured and normalized to 10 pM in accordance with the Illumina MiSeq Denature and Dilute Guide. Paired-end sequencing (2 × 151 bp) was executed on an Illumina MiSeq system using the v3 (600-cycle) reagent kit.

## Results

**Fig. 1:**
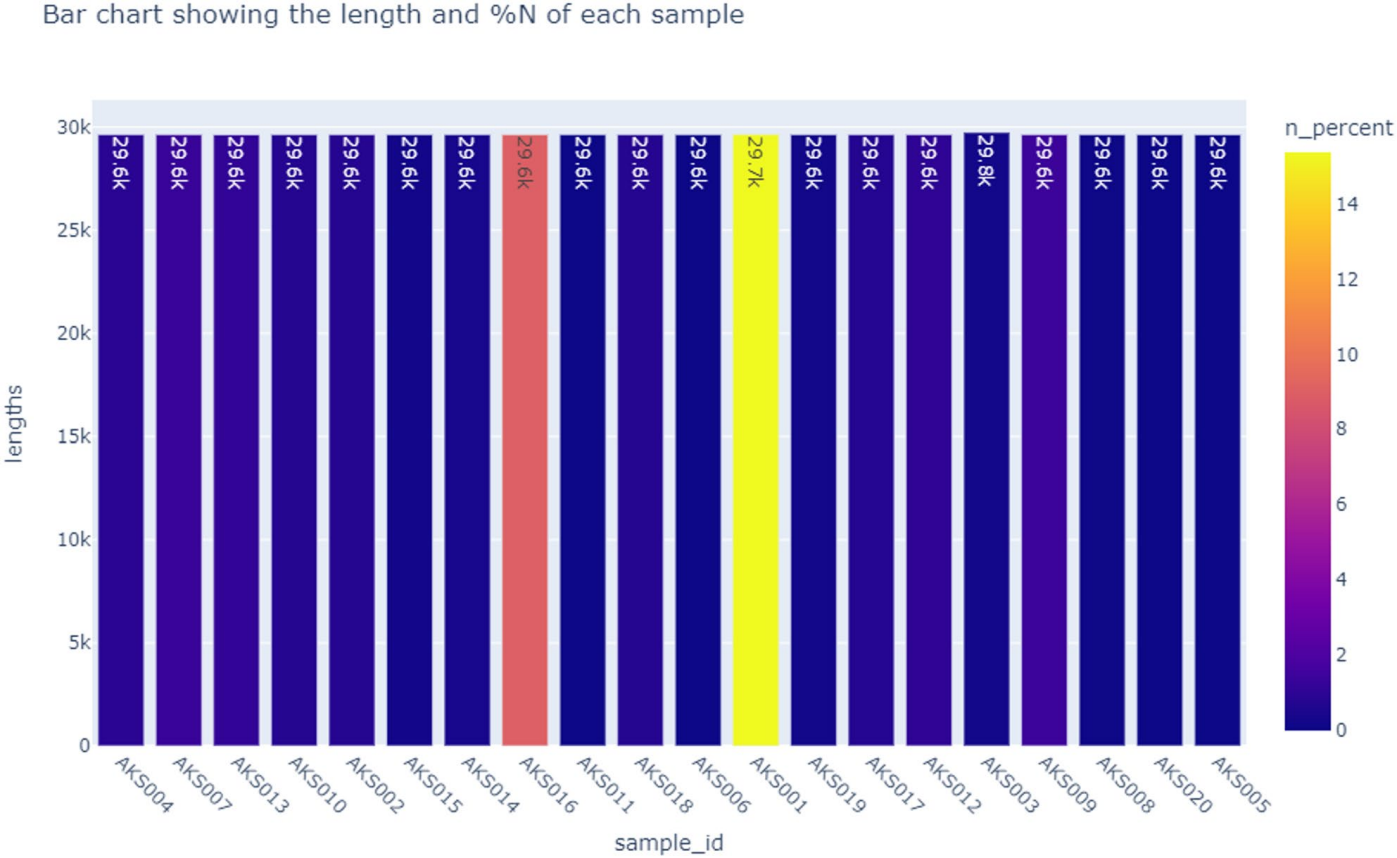
Chart showing the lengths N % of the sequences in relationship with the ambiguous nucleotide percentage

**Fig. 2:**
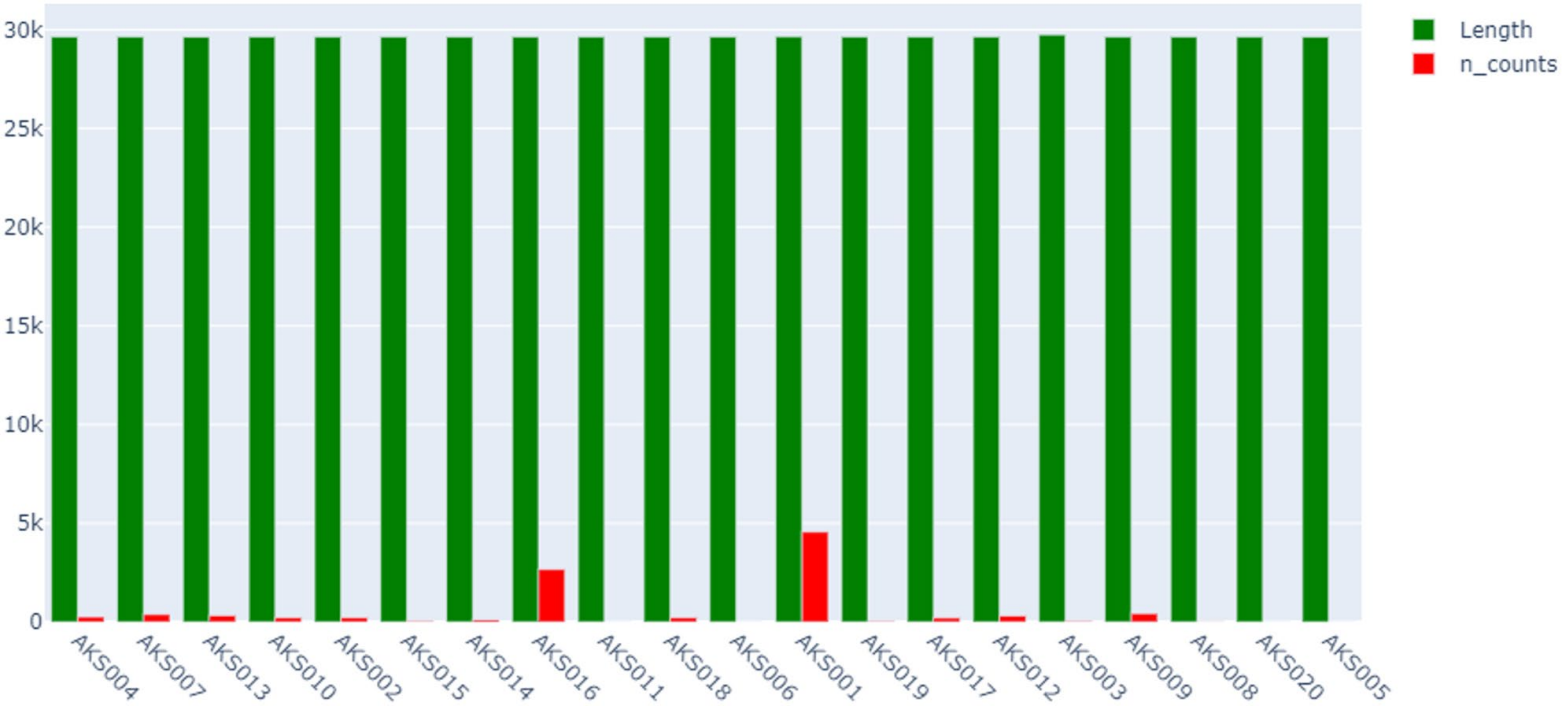
Chart showing the lengths of the sequences in relationship with the ambiguous nucleotides

**Fig. 3:**
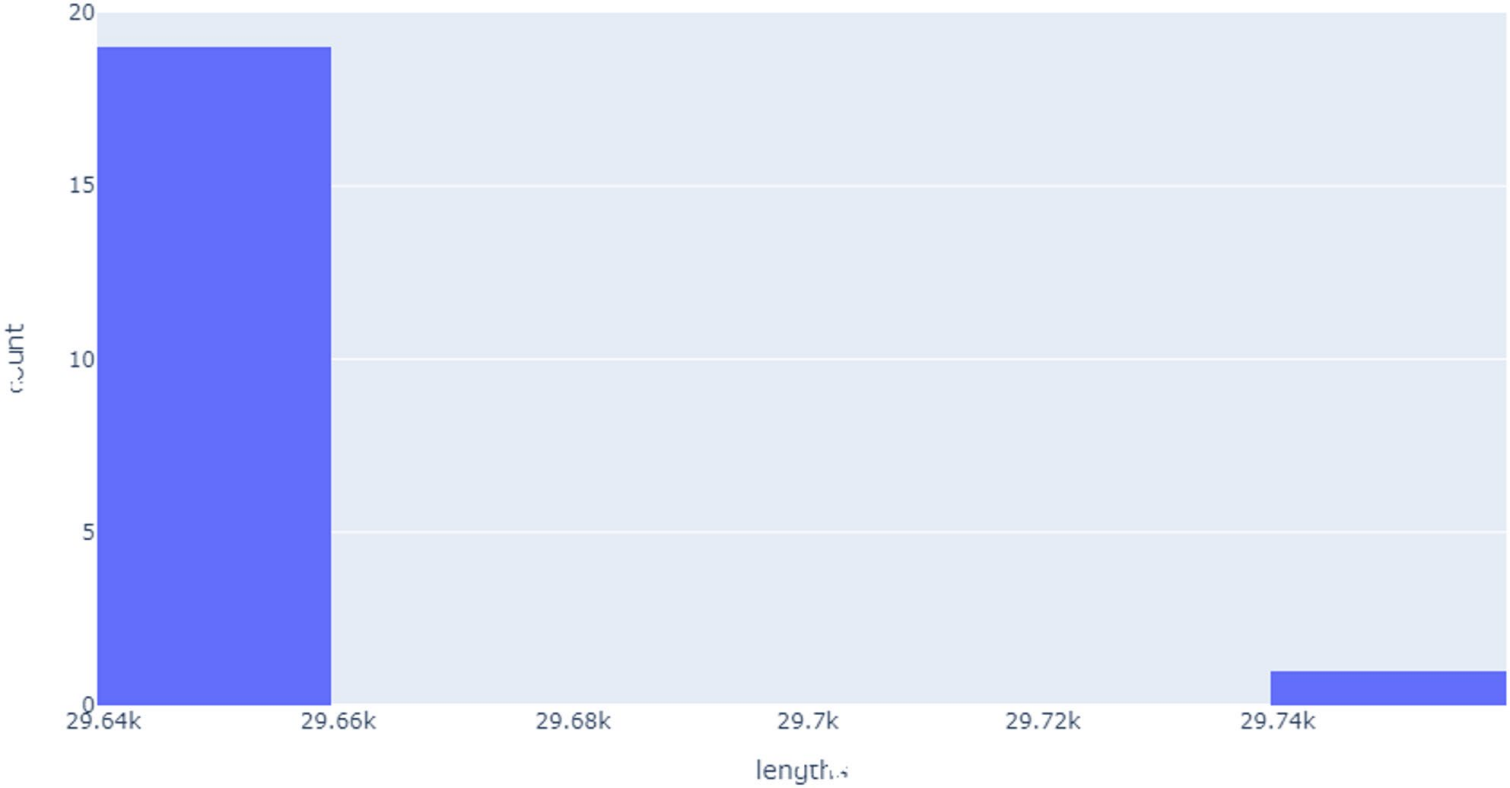
Histogram showing the distribution of the lengths

**Fig. 4:**
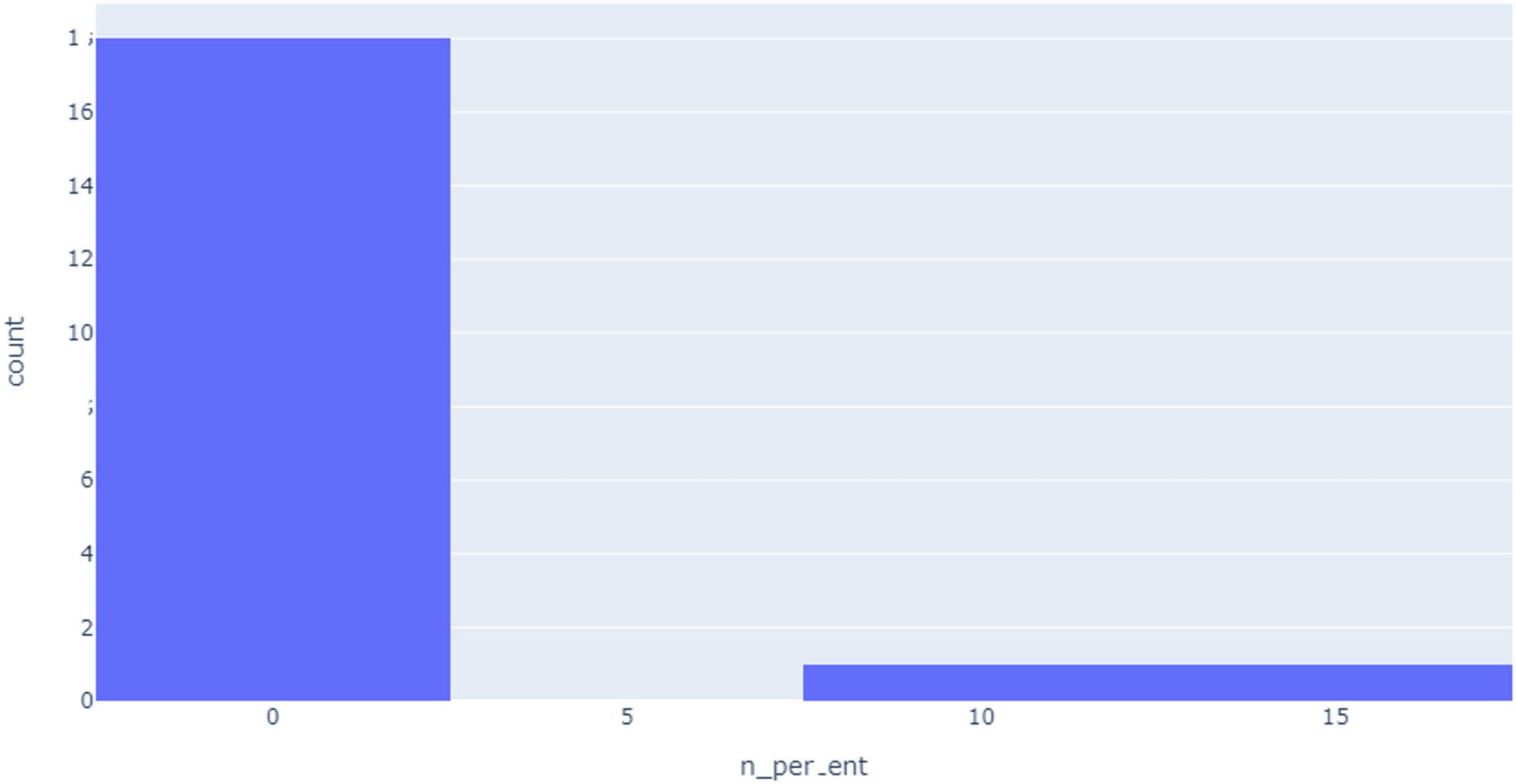
Histogram showing the distribution of the percentage ambiguous nucleotides across sequences

**Fig. 5:**
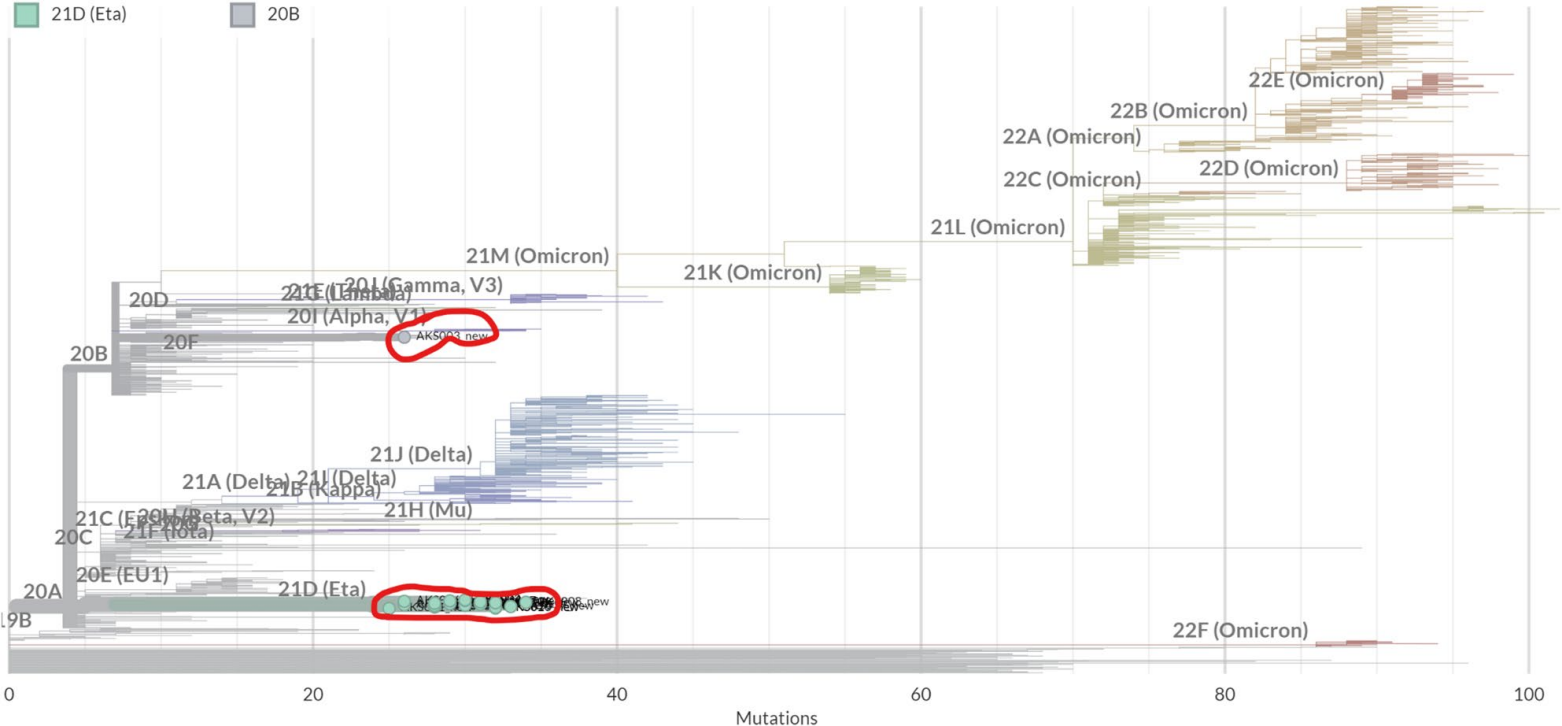
Phylogenetic tree showing the relatedness of the SARS-COV-2 virus strains circulating in Ebonyi State amongst global strains

The sequencing analysis done presents robust genomic lengths with minimal ambiguity (Figs. 1, 2, 3 and 4). The detection of a specific amplicon dropout region and the predominance of clade 21D (Eta) and pangolin lineage B.1.525 among the sequences provide valuable insights into the genomic characteristics and evolutionary trajectory of the SARS-CoV-2 virus (Table 1; Fig. 5). From the result obtained, the genomic analysis of the sequenced samples revealed satisfactory lengths across the genomes, with a notably low frequency of ambiguous nucleotides (N), averaging around 50 per genome. Among the sequences analyzed, AKS003 exhibited the optimal length of 29.8k base pairs (Fig. 1). The result of multiple sequence alignment and amplicon dropout showed that the multiple sequence alignment performed indicates a region of suspected amplicon dropout, spanning positions 2 was 9728 to 29,776. This dropout was unique to this specific region and did not appear elsewhere in the alignment. The result of the lineage assignment and clade distribution performed using NextClade for lineage assignments showed that the majority of the sequences were classified under clade 21D (Eta) and pangolin lineage B.1.525 (Table 1). In total, 11 sequences exhibited less than 2.6% ambiguous nucleotides: thus, qualifying them as full genomes (Figs. 1, 2, 3 and 4). Thus, the NextClade analysis corroborated that most sequences belong to clade 21D (Eta) and pangolin lineage B.1.525. One of the sequences clusters with lineage 2B, while others cluster with lineage 2D (Fig. 5). Genomes of the SARS-CoV-2 in this study have been deposited in GenBank with submission ID: SUB16173024 and assigned GenBank accession numbers: PZ379721-PZ379730.

**Table 1:** COVID-19 sequence analysis report

S/No.	Seq Name	Clade	Nextclade_Pango
1	AKS001	21D (Eta)	B.1.525
2	AKS002	21D (Eta)	B.1.525
3	AKS003	21B	B.1.1.318
4	AKS004	21D (Eta)	B.1.525
5	AKS005	21D (Eta)	B.1.525
6	AKS006	21D (Eta)	B.1.525
7	AKS007	21D (Eta)	B.1.525
8	AKS008	21D (Eta)	B.1.525
9	AKS009	21D (Eta)	B.1.525
10	AKS010	21D (Eta)	B.1.525
11	AKS011	21D (Eta)	B.1.525
12	AKS012	21D (Eta)	B.1.525
13	AKS013	21D (Eta)	B.1.525
14	AKS014	21D (Eta)	B.1.525
15	AKS015	21D (Eta)	B.1.525
16	AKS016	21D (Eta)	B.1.525
17	AKS017	21D (Eta)	B.1.525
18	AKS018	21D (Eta)	B.1.525
19	AKS019	21D (Eta)	B.1.525
20	AKS020	21D (Eta)	B.1.525

## Discussion

The coronavirus disease (COVID-19) initially surfaced in Wuhan, Hubei Province, China, in late December 2019, and its rapid global transmission led to the World Health Organization (WHO) declaring it a pandemic on March 12, 2020 [11]. The virus swiftly spread across continents, causing widespread devastation in numerous countries [12]. By August 2020, the global tally reached 20,836,339 confirmed cases with nearly 750,000 casualties [13]. In Nigeria, the first case emerged on February 28, 2020, and within six months, the number surged to 47,743 cases with 979 deaths as of August 12, 2020 [2]. This study focused on assessing the genetic diversity of the circulating SARS-CoV-2 in Ebonyi State, Southeastern Nigeria. The results in this study revealed a high predominance of SARS-CoV-2 clade 21D (Eta) and pangolin lineage B.1.525 as 19 out of the 20 sequenced strains belonged to this category, while only one belonged to clade 20B and pangolin lineage B.1.1.318. The findings of this study underscore the impact of the SARS-CoV-2 virus at both the global and local levels, emphasizing the need for ongoing research and public health measures to manage and control the spread of COVID-19.

The molecular diagnosis of COVID-19 predominantly relies on detecting the RNA of the SARS-CoV-2 virus [14]. Drawing lessons from previous outbreaks such as SARS-CoV in 2003 and MERS in 2012 [15], a sensitive, specific, and rapid diagnosis for COVID-19 is critical for identifying positive cases, tracing contacts, determining the virus’s source, and implementing effective infection control measures. Early in the epidemic, the complete sequencing of SARS-CoV-2 facilitated specific primer design and laboratory diagnosis of COVID-19 [16].

In Nigeria, the first recorded coronavirus infection in February 2020 was identified in an Italian immigrant. The samples were sent to the African Centre of Excellence for Genomics of Infectious Diseases by the Nigerian Centre for Disease Control for genome sequencing. Globally, there is a singular strain of SARS-CoV-2, which is also the same as the strain circulating in Nigeria. However, there are more than 1,000 lineages of this novel virus circulating worldwide. In this study, the sequencing of the SARS-CoV-2 positive samples revealed good genome length coverage and visibly low ambiguous (N) nucleotides, with most concentrations around 50. The sequence denoted as AKS003 exhibited the best characteristics, with a length of 29,752 and 0 Ns.

Upon conducting a multiple sequence alignment, a region of suspected amplicon dropout was observed from nucleotide position 29,728 to 29,776. This occurrence was singular and not observed elsewhere in the alignment. Further analysis, including NextClade lineage assignments, indicated that the majority of the SARS-CoV-2 (19 strains) belonged to clade 21D (Eta) and the Pangolin lineage B.1.525, while one belonged to clade 21B and the Pangolin lineage B.1.1.318. These lineages are likely to have been selected against by natural evolutionary processes, which aid the understanding of the dynamics of COVID-19 variants.

This information provides valuable insights into the genetic makeup and lineage classification of the sequenced SARS-CoV-2; thus, contributing to a better understanding of the SARS-CoV-2 variants circulating among the populace in Ebonyi State, Southeastern Nigeria.

## Conclusions

This 24-month cross-sectional study conducted from April 2020 to March 2022 in Ebonyi State has provided valuable insights with regard to the circulating SARS-CoV-2 strains within the region. The circulation of SARS-CoV-2 strains belonging to clades 21D (Eta) and 21B and Pangolin lineages B.1.525 and B.1.1.318 respectively contributes to our understanding of the specific viral variants circulating in Ebonyi State. Of significant interest is the high predominance of clade 21D (Eta) and belonging to Pangolin lineage B.1.525 as 19 (95%) out of the 20 sequenced strains belong to this classification. The information in this study will be very impactful in tailoring interventions, adapting response strategies, and preparing for potential challenges posed by specific SARS-CoV-2 strains. Additionally, the enhancement of genomic surveillance through increased sequencing of SARS-CoV-2 strains to monitor evolutionary trends and the emergence of new variants will also be very crucial in understanding the local dynamics of COVID-19; thus, contributing to the broader global effort to combat the pandemic.

## Data Availability

The dataset used and/or analysed in the study is available from the corresponding author upon reasonable request.

## Abbreviations

SARS-CoV-2: Severe Acute Respiratory Syndrome Coronavirus-2
VTM: Viral transport medium
NCDC: Nigeria Centre for Disease Control and Prevention
WHO: World Health Organization
MERS-CoV: Middle East respiratory syndrome coronavirus
